# Co-aerosolized Pulmonary Surfactant and Ambroxol for COVID-19 ARDS Intervention: What Are We Waiting for?

**DOI:** 10.3389/fbioe.2020.577172

**Published:** 2020-09-25

**Authors:** Pradeep Kumar

**Affiliations:** Department of Pharmacy and Pharmacology, Faculty of Health Sciences, School of Therapeutic Sciences, University of the Witwatersrand, Johannesburg, South Africa

**Keywords:** ARDS, COVID-19, pulmonary surfactant, ambroxol, evidence-based therapeutics

## Abstract

After more than 225 days of the first reports of the novel coronavirus from China, COVID-19 pandemic is still on surge. The search for an effective and efficient therapeutic and pharmaceutical intervention is as important and urgent now as it was on Day 1. Majority of the efforts in this direction are toward finding small molecule interventions via repurposing or redirecting the therapeutic approaches. This hypothesis proposes a physical intervention approach directed toward rescuing the complex lung pathology observed in COVID-19 related acute respiratory distress syndrome (CARDS). The loss of content as well as the synthesis and turnover of the surfactant in ARDS has been termed as a “collateral damage.” A synergistic, early stage, cost-effective, pharmaceutically viable, safe, and immediately available solution is hence required. The effectiveness of exogenous surfactant treatment in ARDS has been marred with several limitations as pointed out in various clinical trials and require revised protocols related to surfactant dose and mode of delivery. This hypothesis proposes aerosolized surfactant delivery taking the optimal dosing and coating costs into account along with co-delivery of ambroxol to provide synergistic benefits. Ambroxol is reported to have anti-inflammatory, -oxidant, -viral, and -bacterial activities and has a direct impact on the production and secretion of the surfactant from the alveolar Type 2 cells. If aerosolized, atomized, or nebulized in the form of ambroxol-loaded phospholipid nanovesicles at the early stages of ARDS, depleted surfactant levels may be reinstated and surfactant turnover can be initiated and maintained. The ability to deliver both the components in aerosolized-nebulized form may have a huge impact on alleviating the healthcare burden in low resource settings where the availability of ventilators is limited. In conclusion, the surfactant-ambroxol co-aerosolized intervention approach hypothesized here has implications reaching to clinical and pharmaceutical translation worldwide.

## Introduction: COVID 19 Related ARDS

Since the first confirmed case of COVID-19 in China on 31st December, 2019, the global statistics (at the time of submission of this hypothesis) for confirmed COVID-19 cases stand in excess of 20 million with over 750,000 deaths (Coronavirus Resource Center, 2020 – COVID-19 Dashboard by the Center for Systems Science and Engineering (CSSE) at Johns Hopkins University). This virus has now spread to over 210 countries and territories, and is spreading exponentially within the developing world since mid-March 2020. With the search for effective COVID-19 therapies remain elusive, the management of clinical conditions such as ARDS in COVID-19 patients has become very important. COVID 19 related ARDS (CARDS) has claimed several lives in and out of the ICU and has appeared as a major burden to the healthcare systems across the world (Wu et al., 2020). This burden is further exacerbated by the prolonged stay of the patients in the ICU thereby limiting the availability of healthcare infrastructure and workforce for the newly infected and critically ill patients.

CARDS has been identified to match several characteristics of pre-COVID ARDS at least with respect to (1) the median time from symptom onset to intubation, and (2) the lung physiology (Gibson et al., 2020). On the other hand, in some cases, CARDS fulfilling the Berlin criteria of ARDS is also atypically associated with higher lung compliance wherein hypoxemia may be attributed to the loss of lung perfusion regulation and hypoxic vasoconstriction (Gattinoni et al., 2020b). Based on peculiar phenotypes, Marini and Gattinoni (2020) and Gattinoni et al. (2020a), further classified CARDS as “Type 1: Near normal pulmonary compliance with isolated viral pneumonia/Type L: low lung elastance, lower lung weight, and low response to Positive end-expiratory pressure (PEEP)” and “Type 2: Decreased pulmonary compliance/Type H: high elastance, higher lung weight, and high PEEP response”; and suggested distinctive clinical interventions for both the subtypes. In addition to the above two phenotypes, Zhao et al. (2020), presented an intervening phenotype related to “non-recruitable lungs with low compliance.” The above discussion can further be extended to the known and comprehensive classification of the ARDS (in addition to the Berlin criteria) as reviewed by Calfee et al. (2014), wherein features of the hyperinflammatory and hypoinflammatory ARDS phenotypes were described. It has recently been established that the symptoms and pathology of CARDS may involve a combination of that of viral pneumonia and ARDS. While pulmonary thrombosis is a hallmark of ARDS, diffuse microvascular thrombosis and disseminated intravascular coagulation were distinctively reported in fatal cases of CARDS. This was further accompanied by vascular enlargement, dilated pulmonary vessels (CT scan), and pleuritic pain not typically seen in ARDS. The co-occurrence of pneumonia and ARDS in CARDS may also show distinctive features such as peripheral distribution of opacification and frosted glass opacities. This may be accompanied by round opacities and are now termed as “COVID balls” (Gibson et al., 2020). If screened at an early stage, these unique pathological features may allow for earlier preventative intervention as proposed in this hypothesis.

Although the classification and definition of CARDS is still under investigation, a global consensus on its therapeutic intervention is the need of the hour. In a comment published in The Lancet Respiratory Medicine, Matthay et al. (2020) provided a list of potential evidenced-based therapeutic options for COVID 19 related severe acute respiratory distress syndrome (ARDS) interventions including, but not limited to, high-flow nasal oxygen, positive end-expiratory pressure, neuromuscular blockade, prone positioning, inhaled NO, fluid management, and/or extracorporeal membrane oxygenation. Most recently, Matera et al. (2020) also have provided a narrative review including current challenges and future directions for the pharmacological management of CARDS. This hypothesis is based on previously researched, reviewed, and analyzed studies involving administration of surfactant for ARDS interventions. Additionally, it provides a unique clinical and pharmaceutical perspective targeted at providing therapeutic delivery and physico-chemical interventions for severe ARDS in COVID-19 patients with implications reaching to clinical translation.

## ARDS, Pulmonary Surfactants, and Ambroxol

**FIGURE 1:**
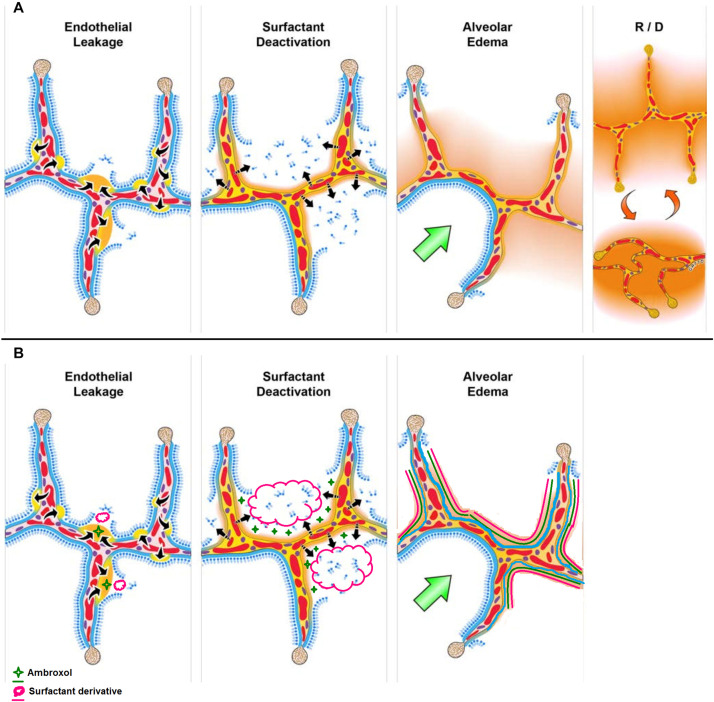
**(A)** The pathologic tetrad of acute respiratory distress syndrome (ARDS). Alveolar walls contain pulmonary capillaries (red circles) and are lined with a liquid hypophase (blue layer inside each alveolus), with an intact pulmonary surfactant layer (small blue ball with tail) layered on the hypophase. The systemic inflammatory response syndrome (SIRS) secondary to sepsis, trauma, burns, pneumonia, and so on increases pulmonary capillary permeability. Endothelial leakage: increased microvascular permeability allowing pulmonary edema to move into the alveolus (black arrows and tan edema blebs). Surfactant deactivation: the continuous layer of pulmonary surfactant molecules is disrupted as the edema blebs expand causing surfactant deactivation (surfactant sluffing off into the alveolar space). Edema usurping surfactant from the alveolar surface, the proteins in the edema fluid deactivating the surfactant, and improper mechanical ventilation causing further surfactant disruption all combine to exacerbate surfactant loss. Alveolar edema: increased capillary permeability and high alveolar surface tension combine to flood alveoli with edema fluid (tan). Recruitment/derecruitment (R/D): loss of surfactant function results in increased alveolar surface tension causing loss of alveolar stability (i.e., causing alveolar R/D with each breath). Alveoli in the top frame of R/D are fully inflated but collapse during expiration in the bottom R/D frame. Alveolar R/D, known as atelectrauma, is another key VILI mechanism. Stress-focus: edema-filled or collapsed alveoli adjacent to air-filled alveoli create a stress-focus causing the alveolar wall to bend toward the fluid-filled alveolus (green arrow), which can cause stress failure at the alveolar wall. Stress-focus is another key mechanism of VILI. Thus, the pathologic tetrad sets up a vicious cycle of high microvascular permeability → edema → surfactant deactivation → high alveolar surface tension → more edema → alveolar R/D → further increase in microvascular permeability → severe ARDS. **(B)** The co-atomized surfactant-ambroxol combination will thus impede the above cycle with reduced inflammation and lowered microvascular permeability → reduced edema → surfactant replenishment and activation → lowered alveolar surface tension → less edema → recovered lung function (Reproduced under Creative Commons Attribution License (CC BY) from Nieman et al. (2020).

In an excellent report published in Frontiers of Physiology, Nieman et al. (2020), described ARDS as a pathologic tetrad with four central components: (1) Endothelial Leakage characterized by increased pulmonary capillary permeability; (2) Surfactant Deactivation resulting into high alveolar surface tension; (3) Alveolar Edema involving flooding alveoli with edema fluid; and finally (4) alveolar recruitment/derecruitment with each breath (Figure 1). It is worth noting that surfactant deactivation occurs quite early in ARDS and the surfactant loss is further exacerbated by the proteins in the edema fluid and by improper ventilation. On the other hand, surfactant loss leads to an increase in surface tension thereby destabilizing the alveolar interface, disrupting the alveolar mechanics, and hence alveoli collapse. The alveolar collapse with limited ventilation further affect the surfactant secretions by type II cells worsening the already reduced surfactant function.

Natural pulmonary surfactant essentially consists of lipids (≈90%) and proteins (≈8–10%). Phospholipids such as dipalmitoylphosphatidylcholine (DPPC) form the major part of the lipids while neutral lipids account for 5–10%. The protein part of the surfactant is composed of two different types: hydrophilic (SP-A and SP-D) proteins from the collectin family and hydrophobic apolipoproteins (SP-B and SP-C). The bio-chemo-physical function of the above components is very complex. The phospholipid component is mainly responsible for the low surface tension due to its high packing within the interfacial surfactant films. Negatively charged phospholipids such as phosphatidylglycerol (PG) and phosphatidylinositol (PI), although available in low quantities, play a crucial role in interacting with the cationic hydrophobic proteins (SP-B and SP-C). These interactions are essential for the interfacial transfer of the lipids and for the formation of a surface-active film. SP-A and SP-D play a more biochemical function by providing innate immune defense (Pérez-Gil, 2008; Echaide et al., 2017). Further to this, Echaide et al. (2017) comprehensively defined and described the composition and importance of pulmonary surfactant as “Pulmonary surfactant is a complex of lipids and proteins assembled and secreted by the alveolar epithelium in to the thin layer of fluid coating the respiratory surface of lungs. There, surfactant forms interfacial films at the air water interface, reducing dramatically surface tension and thus stabilizing the air-exposed interface to prevent alveolar collapse along respiratory mechanics. The absence or deficiency of surfactant produces severe lung pathologies.”

Surfactant replacement therapy for adult ARDS have been the subject of several clinical trials and meta-analyses. Most recently, Meng et al. (2019) reported a meta-analysis of randomized controlled trials related to surfactant administration in adult ARDS (RCTs: 11, patients: 3038, and surfactants: Exosurf/Venticute/Pneumasurf/HL-10/natural extracts) and listed the mechanism of action of pulmonary surfactant in ARDS as (1) maintenance of lower alveolar tension and stabilization of the alveolar volume, (2) promotion of gas exchange and distribution, (3) reduction of edema in alveoli and interstitium, (4) modulation of systemic inflammatory reactions, and (5) reduction of local mechanical forces (Meng et al., 2019). Davidson et al. (2006) (RCTs: 06, patients: 1323, and surfactants: Exosurf/Survanta/Venticute), reported that exogenous surfactant intervention may improve oxygenation but may not have significant effect on the mortality outcome. On the other hand, Zhang et al. (2013) (RCTs: 07, patients: 2144, and surfactants: Exosurf/Venticute/natural extracts), couldn’t “accurately define whether exogenous surfactant has an effect on oxygenation from the included studies.” Finally, Dushianthan et al. (2012) (RCTs: 08, patients: 2615, and surfactants: Exosurf/Survanta/Venticute/natural extracts), provided a clinical review of exogenous surfactant for ARDS intervention. The review report established that the conventional synthetic surfactant replacement approach may have some benefits for oxygenation improvement but the survival benefits were not conclusive. Some mortality benefits were reported when a recombinant SP-C based surfactant was administered in patients with ARDS induced by aspiration and pneumonia. The study further highlighted the need to innovate and optimize the composition of the surfactant to obtain a natural mimic especially by incorporating the right mix of surfactant proteins (Dushianthan et al., 2012). Due to small sample size of the chosen trials and the inconsistent/conflicting findings of individual studies, these reports suggested that large rigorous clinical trials need to be conducted to reach a definite outcome.

Ambroxol [2-amino-3,5-dibromo-N-(trans-4-hydroxycyclohexyl)benzylamine], a metabolite of bromhexine, is an over the counter mucolytic agent and has been clinically used for various respiratory disorders over the last 40 years (Kantar et al., 2020). In addition to being a clinically proven mucoactive agent and a secretagogue, ambroxol is reported to have anti-inflammatory, anti-oxidant, anti-viral, and anti-bacterial properties (Malerba and Ragnoli, 2008, Paleari et al., 2011). For the purpose of current application; it is worth noting that high dose ambroxol (≥15 mg/kg or 1,000mg/day) has previously been employed for ARDS intervention and has been reported to improve PaO_2_/FiO_2_, PO_2_, and SaO_2_ as well as an improvement in the phospholipid profile of tracheal effluent in ARDS patients (Wu et al., 2014). In addition, the meta-analysis concluded that high-dose ambroxol treatment has been reported to reduce SOD, TNF-α, and IL-6 levels in the serum as well as reduction in acute contusion of lung and the ICU stay (Wu et al., 2014). Furthermore, ambroxol has shown high affinity toward the lung tissue (16 times higher than that of the serum) and may stay for over 8 h at this level (Mezzetti et al., 1990). This prolonged presence of ambroxol is capable of stimulating phospholipid synthesis as well as play a major role in generation and secretion of pulmonary surfactant (Xiang and Wang, 2019). Most importantly, the inactivation of surfactant by reactive oxygen species particularly involves both structural and functional alterations to SP-B and SP-C (Rodríguez-Capote et al., 2006). It is worth noting that ambroxol not only is capable of modulating surfactant production from alveolar cells but also increases the expression levels of SP-B and SP-C and hence may contribute to the interfacial turnover of the surfactant as explained above under the surfactant composition (Seifart et al., 2005; Kanie et al., 2017).

## The Hypothesis and the Discussion

Several therapeutics agents such as steroidal drugs, antiretrovirals, and antimalarial drugs are under investigation and trial; and several others are being repurposed for COVID 19 intervention (Zhou et al., 2020). In addition, Rice and Janz (2020) provided an inspiring account of excellent, evidence-based, interventional recommendations related to ventilation support for CARDS and discussed related clinical features. Oxygen and ventilation interventions may assist with the much needed respiratory compliance, but may not address the underlying physicochemical aspects such as building up of mucous and alveolar tension. This hypothesis proposes the evidence-based use of inhalable, aerosolized, natural or exogenous pulmonary surfactants to (1) act as therapeutic agents; and (2) to act as nanocarriers (vesicles) or aerosolizing agents for co-administered drugs such as ambroxol (or other drugs under clinical use).

From Figure 1, it is clear that ARDS involves more than just the absence of the surfactant as in the case of NRDS. Several RCT studies conducted for surfactant intervention in ARDS had resulted in modest and transient outcomes. This may be due to the complex pathology of ARDS (as compared to NRDS) including inherent loss and/or inactivation of the endogenous and/or exogenous pulmonary surfactant (Taeusch et al., 2005). Willson (2015), justifiably described the role of surfactant loss and deactivation of surfactant synthesis and turnover in the complex pathology in non-neonatal RDS as “collateral damage.” This further informs us that the surfactant intervention approach for ARDS needs to be different from that of NRDS. Corroborating the above notion, Raghavendran et al. (2011), highlighted the limitations of currently employed commercially available surfactants (Exosurf^®^ and Survanta^®^) as well as the lack of targeted pulmonary surfactant interventions in ARDS. With CARDS being a direct pulmonary form of ARDS, delivery of an exogenous surfactant directly to the alveoli is warranted. The authors further stressed on combination therapy approaches (targeted to alveoli or even intravenous) alongside surfactant therapy to attain improved outcomes (see Table 8 of the reference). Furthermore, taking lessons from NRDS, surfactant therapy can be augmented with physical approaches such as nasal continuous positive airway pressure (nCPAP) (Verder et al., 2009).

In a correspondence published in the American Journal of Respiratory and Critical Care Medicine, the authors raised concerns about the suboptimal dose of instilled surfactant and hence the alveolar delivery and recommended that the “coating cost” (surfactant required to coat the conducting airway tree) should be taken into account (Grotberg et al., 2017). An adult lung has ≈4,500 cm^2^ of conducting airway surface leading to significant surfactant loss before it reaches the alveoli. This means that not only dose but the method of delivery also becomes a limiting factor. Once the dose is finalized and accounted for, the delivery of the aerosolized surfactant can be achieved via different approaches in and out of the ICU such as, but not limited to, non-invasive nebulization, CPAP, and NIV; and, if required, using invasive intubated ventilation (Shah, 2011; Willson, 2015; Dhand, 2017; Rzepka-Wrona et al., 2018). Although these clinical techniques are well known and employed, targeted nanocarriers capable of self-demicellization may provide the strategy to cover or even remove the coating cost. However, targeting strategies may complicate the formulation of the components and may further add to costs.

But not all is doom and gloom in case of surfactant therapy in ARDS. As early as in 1993, Lewis and Jobe, reported positive results – improved gaseous exchange and a decreased mortality trend – from a randomized clinical trial which evaluated the efficacy of aerosolized exogenous surfactant in ARDS. Based on the results, the authors suggested development of optimal surfactant delivery techniques as well as of optimal exogenous surfactant preparations (Lewis and Jobe, 1993). This can be achieved by atomization of the pulmonary surfactant using standardized and clinically relevant approaches. Coming to the efficient and targeted pulmonary delivery of various aerosolizable agents, Dhand (2017), published a seminal report describing the goals of inhalation therapy during mechanical ventilation as targeted delivery assuring and optimizing drug deposition in the lung; consistent and reproducible dosing; clinically feasible and safe delivery of inhaled drugs; and cost-effectiveness (Willson, 2015).

Where applicable, to affect the efficient and effective localized pulmonary delivery of such drugs; the second aspect of this hypothesis is based on the inherent amphiphilic and hence self-assembling properties of exogenous pulmonary surfactant components (such as dipalmitoylphosphatidylcholine; DPPC) to form liposomes and vesicles. Due to the amphiphilic nature of pulmonary surfactants, drugs and therapeutics of varying solubility and solid-state properties can be incorporated and/or coacervated into these carriers by “simple” admixing of the drug with the aerosolized surfactant (Yan et al., 2012). DPPC has been well researched and employed for developing liposomal inhalation suspensions for targeted lung delivery and interventions including penetration of biofilms and uptake into macrophages (Chimote and Banerjee, 2010; Zhang et al., 2018). In addition, endogenous lung surfactant inspired strategies including phospholipid based nanovesicle aerosols (Joshi et al., 2015; Altube et al., 2017; Parra et al., 2018) have been reported as pulmonary compatible and efficient drug delivery platforms.

An optimized exogenous surfactant combination with the right components for mimicking the natural pulmonary surfactant is urgently needed to be considered including from the group of known and in practice ones such as Surfacen, Infasurf, Survanta, Curosurf, Exosurf, Venticute, Pneumasurf, HL-10, and even natural extracts. Most recently a promising synthetic recombinant SP-C analog (SPC33Leu) has been proposed in a published thesis from Karolinska Intitutet (Basabe-Burgos, 2020). Given the urgency of the current pandemic, designing and regulatory approval of new surfactants will be a lengthy process but may still qualify for a clinical trial. There may not be direct data and evidence as yet for the surfactant levels in patients with CARDS; but there are confirmatory biopsies delineating toward the damage of alveolar Type II cells which essentially are involved in surfactant production in the lungs (Tian et al., 2020; Xu et al., 2020).

Ambroxol as an adjuvant to pulmonary surfactant intervention is an important proposition here. As described by Plomer and de Zeeuw (2017), ambroxol is more than just an expectorant and is a potent inducer of surfactant synthesis from alveolar Type 2 cells (Plomer and de Zeeuw, 2017; Depfenhart et al., 2020). In addition to this, ambroxol may also assist in the secretion of surfactant lipids by the Type 2 cells (Post et al., 1983). This is an important consideration given the “collateral damage” that occurs to pulmonary surfactant levels and synthesis as a result of ARDS.

A co-aerosolized, DPPC-ambroxol intervention may present a synergistic outcome in ARDS intervention at early stages and may be a potential clinical trial candidate for COVID-19 as it may essentially provide with alleviation of the inherent inflammatory response, attenuation of epithelial cell damage, decreased interstitial exudation and edema, and hence reduced lung damage. When co-administered, ambroxol may act by reducing the inflammation (Takeda et al., 2016; Kanie et al., 2017) which in turn may minimize the pulmonary capillary permeability thereby affecting the edema blebs (Figure 1). Concurrently, the exogenous surfactant delivered to the lungs may replenish the deactivated surfactant and hence the surfactant lining will be restored. From a formulation and pharmaceutical perspective, this DPPC-ambroxol hypothesis is further substantiated by the aerosolizability and nebulizability of ambroxol (Paleari et al., 2011). The final formulation may be developed as an “atomized, nebulized, DPPC nanovesicles coated- and loaded- with ambroxol.” The coating of nanovesicles with ambroxol is important given the binding of ambroxol to the lung lining as explained above. Since the above system may act as a nanocarrier, adjuvant drugs such as dexamethasone can also be loaded and delivered to the lungs as a multitherapeutic-multifunctional nanosystem thereby reducing their systemic side effects.

The outcomes and activities from the European COST Action MP1404 “SimInhale – Simulation and Pharmaceutical Technologies for Advanced Patient-Tailored Inhaled Medicines” may provide the essential and important clinical, pharmaceutical, and industrial applicability and translation guidance for the hypothesis (Fattal and Kassinos, 2018). If delivered effectively, with right dosage, in a timely manner, and in combination with other therapeutic agents; this early intervention, pending trials, may save many lives and may even assist in reducing burden on the healthcare system.

## Conclusion

A novel and innovative physical therapeutic intervention approach is hereby proposed for COVID-19 related ARDS. Co-aerosolized exogenous pulmonary surfactant and ambroxol may provide synergistic benefits related to repletion and coating of pulmonary linings with surfactant as well as alleviation of various surfactant abnormalities (reduced surfactant synthesis and recycling and the ROS induced oxidative stress) by ambroxol. This approach is easily translatable to a pharmaceutical and clinical setting; and have the potential to be considered for an urgent and immediate COVID-19 clinical trial. In addition, this approach will provide the scientific community some clarity on the effectiveness of both the components for ARDS intervention – a conundrum troubling the scientific community for decades.

## Meta-Analyses Included in This Hypothesis and Relevant Table Citation Referred Herein

Davidson et al. (2006): Exogenous pulmonary surfactant for the treatment of adult patients with acute respiratory distress syndrome (Table 2).

Dushianthan et al. (2012): Exogenous surfactant therapy for acute lung injury/acute respiratory distress syndrome (Table 5).

Zhang et al. (2013): Exogenous pulmonary surfactant for acute respiratory distress syndrome in adults (Table 1).

Meng et al. (2019): Effect of surfactant administration on outcomes of adult patients in acute respiratory distress syndrome (Table 2).

## Author Contributions

PK conceived the hypothesis, and wrote and discussed the hypothesis.

## Conflict of Interest

The author declares that the research was conducted in the absence of any commercial or financial relationships that could be construed as a potential conflict of interest.

## Dedication

This article is dedicated to Prof. Viness Pillay (1970–2020), Wits University, who tragically passed away on 24th July, 2020.
